# Iniquidades ambientais na metrópole paulista, Brasil: vegetação, calor e poluição do ar segundo a vulnerabilidade social

**DOI:** 10.1590/0102-311XPT177725

**Published:** 2026-07-20

**Authors:** Claudio Makoto Kanai, Rafael Buralli, Luis Roberto da Silva, Nelson Gouveia

**Affiliations:** 1 Faculdade de Medicina da Universidade de São Paulo, São Paulo, Brasil.; 2 Faculdade de Saúde Pública, Universidade de São Paulo, São Paulo, Brasil.

**Keywords:** Saúde Ambiental, Saúde Urbana, Iniquidades em Saúde, Justiça Ambiental, Indicadores Ambientais, Environmental Health, Urban Health, Health Inequities, Environmental Justice, Environmental Indicators, Salud Ambiental, Salud Urbana, Inequidades en Salud, Justicia Ambiental, Indicadores Ambientales

## Abstract

As desigualdades socioambientais nas metrópoles afetam a saúde de forma diferente, sobretudo com impactos maiores entre populações de menor nível socioeconômico. Este estudo analisou a distribuição da vegetação urbana, estimada pelo Índice de Vegetação por Diferença Normalizada (NDVI), da temperatura de superfície terrestre (TST) e das concentrações de dióxido de nitrogênio (NO_2_) na Região Metropolitana de São Paulo, Brasil, segundo grupos de vulnerabilidade definidos pelo Índice Paulista de Vulnerabilidade Social (IPVS). Trata-se de estudo ecológico tendo os setores censitários urbanos como unidade de análise. A vegetação e a TST foram obtidas por sensoriamento remoto, e o NO_2_ derivou de modelo global em alta resolução baseado em regressão de uso do solo. Resumos estatísticos compararam os indicadores entre os grupos do IPVS (1 - baixíssima a 6 - muito alta vulnerabilidade). As medianas do NDVI variaram pouco (0,21-0,25), sem padrão linear; o grupo de alta vulnerabilidade apresentou valores mais elevados, possivelmente pela localização periurbana. Para a TST, o grupo de baixíssima vulnerabilidade registrou a menor mediana (31,8ºC). O NO_2_ apresentou tendência de queda entre os grupos menos vulnerabilizados até o de alta vulnerabilidade, com novo aumento no de muito alta vulnerabilidade. Conclui-se que a exposição ambiental na Região Metropolitana de São Paulo é heterogênea e não segue padrão linear em relação à vulnerabilidade social. Particular atenção deve ser dada às áreas mais vulnerabilizadas que acumulam condições ambientais críticas, reforçando a necessidade de políticas públicas integradas para reduzir desigualdades e promover cidades mais saudáveis e equitativas.

## Abstract (en)

Socio-environmental inequalities in large cities affect health in different degrees, more greatly impacting poorer populations. This study analyzed the distribution of urban vegetation (estimated by the Normalized Difference Vegetation Index - NDVI), land surface temperature (LST), and nitrogen dioxide concentrations (NO_2_) in the Metropolitan Area of São Paulo, Brazil, according to vulnerability groups defined by the São Paulo Social Vulnerability Index (IPVS). Urban census tracts were used as the unit of analysis in this ecological study. The NDVI and LST were obtained by remote sensing and NO_2_ were derived from a high-resolution global model based on land use regression. Statistical summaries compared the indicators between the IPVS groups (1 - very low to 6 - very high vulnerability). NDVI medians scarcely varied (0.21-0.25) and showed no linear pattern; the high vulnerability group had higher values (possibly due to their peri-urban location). The group with very low vulnerability showed the lowest LST median (31.8ºC). NO_2_ concentrations showed a downward trend between the less and highly vulnerable groups, with a further increase in the latter. The Metropolitan Area of São Paulo shows heterogeneous environmental exposure without a linear pattern regarding social vulnerability. The most vulnerable areas accumulating critical environmental conditions require particular attention, reinforcing the need for integrated public policies to reduce inequalities and promote healthier and more equitable municipalities.

## Resumen

Las desigualdades socioambientales en las áreas metropolitanas afectan la salud de manera diferente, especialmente con mayores repercusiones entre las poblaciones de menor nivel socioeconómico. Este estudio analizó la distribución de la vegetación urbana, estimada por el Índice de Vegetación de Diferencia Normalizada (NDVI), de la temperatura de la superficie terrestre (TST) y de las concentraciones de dióxido de nitrógeno (NO_2_) en la Región Metropolitana de São Paulo, Brasil, según los grupos de vulnerabilidad definidos por el Índice Paulista de Vulnerabilidad Social (IPVS). Se trata de un estudio ecológico que utiliza las zonas censales urbanas como unidad de análisis. La vegetación y la TST se obtuvieron mediante teledetección, y el NO_2_ se derivó de un modelo mundial de alta resolución basado en la regresión del uso del suelo. Los resúmenes estadísticos compararon los indicadores entre los grupos IPVS (1 - bajísima a 6 - muy alta vulnerabilidad). Las medianas del NDVI variaron poco (0,21-0,25), sin un estándar lineal; el grupo de alta vulnerabilidad mostró valores más altos, posiblemente debido a su ubicación periurbana. En el caso de la TST, el grupo con bajísima vulnerabilidad registró la mediana más baja (31,8ºC). El NO_2_ presentó una tendencia a la baja entre los grupos menos vulnerables y el grupo de alta vulnerabilidad, con un nuevo aumento en el grupo de muy alta vulnerabilidad. Se concluye que la exposición ambiental en la Región Metropolitana de São Paulo es heterogénea y no sigue un estándar lineal en relación con la vulnerabilidad social. Se debe prestar especial atención a las zonas más vulnerables que acumulan condiciones ambientales críticas, lo que refuerza la necesidad de políticas públicas integradas para reducir las desigualdades y promover ciudades más saludables y equitativas.

## Introdução

Com o rápido crescimento da população mundial, a saúde humana e o desenvolvimento de ambientes saudáveis têm sido impactados desigualmente, afetando de maneira mais intensa as comunidades vulnerabilizadas, como aquelas com menor nível socioeconômico ^1^. Uma das consequências é a redução ou a escassez de áreas verdes no ambiente urbano, que contribui para o aumento das temperaturas e a deterioração da qualidade do ar, impactando negativamente a saúde física e mental da população ^2^. Evidências apontam que ampliar áreas verdes nos centros urbanos pode melhorar a qualidade de vida e a saúde dos indivíduos ^3^. Realizar tal intervenção é relevante, principalmente baseadas na lógica de justiça ambiental, priorizando locais ondem vivem as populações mais vulnerabilizadas ^4^.

Na Região Metropolitana de São Paulo, Brasil, a urbanização acelerada e a falta de planejamento e infraestrutura urbana consolidaram territórios de contrastes, com sobreposição espacial entre vulnerabilidade social e riscos ambientais ^5^, tornando a região estratégica para investigar iniquidades ambientais e em saúde. Portanto, este trabalho objetiva descrever a cobertura de áreas verdes, temperatura e níveis de poluição do ar nos setores censitários da Região Metropolitana de São Paulo, segundo categorias de vulnerabilidade social.

## Métodos

Trata-se de um estudo ecológico, tendo como unidade de análise os setores censitários urbanos da Região Metropolitana de São Paulo do *Censo Demográfico* de 2010 ^6^. A exposição ambiental foi estimada nos territórios analisados calculando-se as médias do Índice de Vegetação por Diferença Normalizada (NDVI, acrônimo em inglês), da temperatura da superfície terrestre (TST) e das concentrações de dióxido de nitrogênio (NO_2_).

O NDVI foi calculado com base em imagens Landsat 8 (resolução de 30m) obtidas no EarthExplorer (https://earthexplorer.usgs.gov/), usando-se as bandas 4 (vermelho) e 5 (infravermelho próximo). Foram selecionadas imagens do período seco: 02/Jul/2014, 03/Ago/2014, 23/Set/2015 e 07/Jul/2016. O índice foi estimado no QGIS 3.34.10 (https://qgis.org/en/site/), pela ferramenta *Calculadora Raster*, aplicando-se a fórmula padrão:

$$NDVI=\frac{\left(Banda5-Banda4\right)}{\left(Banda5+Banda4\right)}$$

Os valores de NDVI variam de -1 a +1: próximos de 1 indicam maior cobertura vegetal; próximos de 0, áreas construídas ou solo exposto; e negativos, corpos d’água ou nuvens ^7^.

A TST foi estimada baseando-se na banda 10 termal do Landsat 8 (30m), obtida no EarthExplorer, em dias de maior calor: 08/Fev/2014, 10/Jan/2015 e 16/Fev/2017. O processamento foi feito no QGIS (*plugin Semi-Automatic Classification* ^8^), que converte os dados espectrais em graus Celsius (ºC).

As concentrações de NO_2_ vieram de estimativas globais em alta resolução (50m) de Larkin et al. ^9^, baseadas em modelo de regressão com dados de médias diárias de monitores de qualidade do ar, densidade da coluna troposférica de NO_2_ obtidas por sensoriamento remoto, infraestrutura viária, ambiente construído e meteorologia.

As médias de NDVI, TST e NO_2_ foram calculadas por setor censitário, excluindo locais sobre massas d’água para evitar subestimação. Os limites dos corpos hídricos, obtidos do Global Surface Water Explorer (https://global-surface-water.appspot.com/) ^10^, foram usados no recorte, pois essas áreas apresentam temperaturas mais baixas e NDVI negativos.

A vulnerabilidade social foi classificada pelo Índice Paulista de Vulnerabilidade Social (IPVS) da Fundação Sistema Estadual de Análise de Dados (SEADE) ^11^, construído com indicadores socioeconômicos e demográficos do *Censo Demográfico* de 2010. O índice categoriza os setores urbanos em seis níveis: 1 (baixíssima), 2 (muito baixa), 3 (baixa), 4 (média), 5 (alta) e 6 (muito alta). Utilizou-se a versão de 2010 pela indisponibilidade de atualização para 2022 até o encerramento deste estudo.

A distribuição dos indicadores ambientais por grupos do IPVS foi descrita por medianas e intervalos interquartis (IIQ) para NDVI, TST e NO_2_. As análises descritivas e a elaboração dos gráficos foram realizadas no software Jamovi, versão 2.3.28 (https://www.jamovi.org), e R, versão 4.4.0 (http://www.r-project.org), e os mapas no software QGIS.

## Resultados

**Figura 1:**
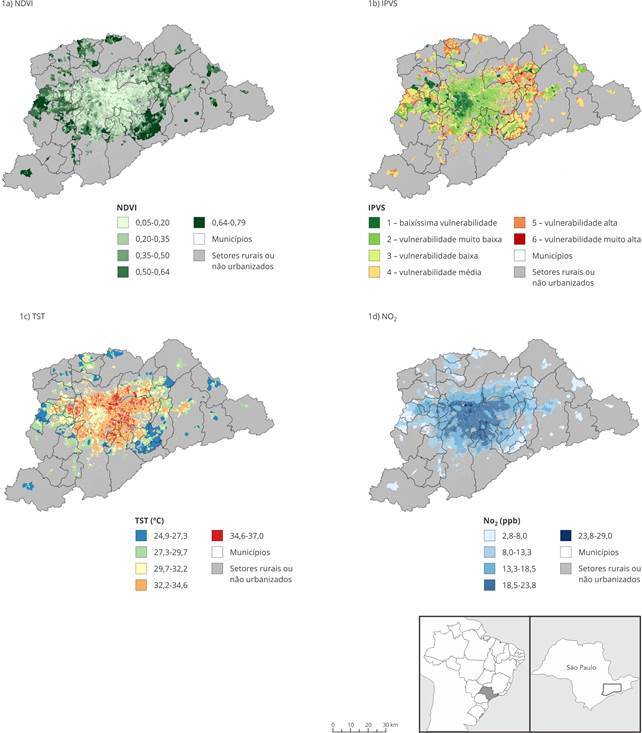
Mapas com a distribuição espacial de Índice de Vegetação por Diferença Normalizada (NDVI), temperatura da superfície terrestre (TST) e dióxido de nitrogênio (NO_2_), por setor censitário, na Região Metropolitana de São Paulo, Brasil.

A Região Metropolitana de São Paulo apresentou heterogeneidade na distribuição dos indicadores ambientais. A Tabela 1 resume as estatísticas descritivas dos 27.630 setores censitários, tanto para o conjunto global quanto estratificadas pelos grupos do IPVS, e a Figura 1 ilustra a distribuição espacial dos indicadores ambientais e dos níveis de vulnerabilidade social. O NDVI apresentou mediana de 0,22 (IIQ: 0,18-0,28), a TST de 33,4ºC (IIQ: 32,1-34,2) e o NO_2_ de 17,50ppb (IIQ: 14,90-19,59) (Tabela 1).

**Tabela 1:** Resumo estatístico das variáveis ambientais na Região Metropolitana de São Paulo, Brasil.

Variável/Grupo do IPVS	Mediana	Mínimo	Máximo	1º quartil	3º quartil
NDVI					
Global	0,22	0,05	0,79	0,18	0,28
1	0,23	0,06	0,73	0,19	0,30
2	0,21	0,05	0,75	0,18	0,25
3	0,22	0,06	0,77	0,18	0,28
4	0,22	0,08	0,79	0,19	0,29
5	0,25	0,09	0,79	0,20	0,37
6	0,21	0,09	0,74	0,18	0,26
TST (ºC)					
Global	33,39	24,90	36,99	32,13	34,18
1	31,75	25,50	35,48	30,73	32,68
2	33,85	25,48	36,48	33,11	34,40
3	33,36	25,23	36,33	32,26	34,18
4	33,47	24,90	36,30	32,19	34,24
5	32,46	25,59	36,43	30,72	33,64
6	33,32	26,69	36,99	32,06	34,18
NO_2_ (ppb)					
Global	17,50	2,75	29,00	14,90	19,59
1	20,50	4,91	28,75	18,56	22,00
2	18,32	3,78	28,67	16,35	19,89
3	16,72	3,51	29,00	14,04	18,83
4	16,00	3,67	25,28	13,21	18,00
5	14,47	2,75	24,95	11,61	16,83
6	16,60	6,93	24,50	14,00	18,58

IPVS: Índice Paulista de Vulnerabilidade Social; NDVI: Índice de Vegetação por Diferença Normalizada; TST: temperatura da superfície terrestre.

Fonte: elaboração própria.

Nota: grupos do IPVS: 1 - baixíssima vulnerabilidade; 2 - muito baixa; 3 - baixa; 4 - média; 5 - alta; 6 - muito alta. Número de setores por grupo do IPVS: 1 = 3.407; 2 = 10.678; 3 = 4.782; 4 = 4.234; 5 = 2.638; 6 = 1.891 (total = 27.630 setores).

IPVS: Índice Paulista de Vulnerabilidade Social.

**Figura 2:**
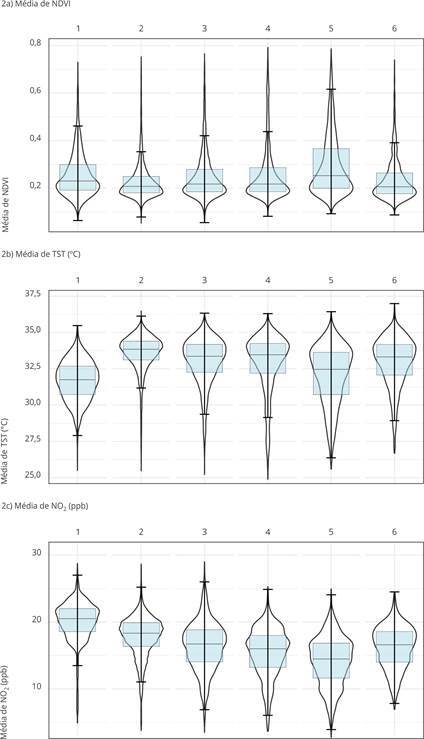
*Boxplots* e gráficos de violinos da distribuição de médias de Índice de Vegetação por Diferença Normalizada (NDVI), temperatura da superfície terrestre (TST) e dióxido de nitrogênio (NO_2_) entre grupos do Índice Paulista de Vulnerabilidade Social (IPVS).

A análise das variáveis ambientais por grupos do IPVS revelou padrões distintos de exposição e variabilidade dos dados intragrupos, evidenciada pelas amplitudes de cada indicador (Figura 2).

A distribuição do NDVI não apresentou relação linear com a vulnerabilidade social. As medianas variaram pouco entre os grupos (0,21-0,25), indicando diferenças pouco expressivas na cobertura vegetal. Os grupos 2 e 6 registraram as menores medianas (0,21). O grupo 5 apresentou a maior mediana (0,25) e terceiro quartil mais elevado (0,37), também refletida pela cauda mais longa nos diagramas de violino. O grupo 1 registrou a segunda maior mediana de 0,23 e ampla dispersão (0,06-0,73), revelando heterogeneidade de cobertura vegetal mesmo em áreas menos vulnerabilizadas. Já os grupos 3 e 4, com mediana de 0,22, exibiram distribuições semelhantes (Tabela 1).

Quanto à TST, os grupos mais vulnerabilizados apresentaram valores mais altos que o grupo 1, que registrou a menor mediana (31,8ºC). Os grupos 2, 3, 4 e 6 registraram medianas elevadas, destacando-se o grupo 2 pela maior mediana e concentração de valores, indicada pelo menor IIQ (33,1-34,4) e pelo diagrama de violino. O grupo 5 aproximou-se do grupo 1 na mediana, mas exibiu distribuição mais alongada entre o mínimo e primeiro quartil, indicando maior variabilidade nas temperaturas baixas (Tabela 1).

A distribuição de NO_2_ mostrou padrão inverso à vulnerabilidade social, com maiores concentrações nos grupos menos vulnerabilizados. O grupo 1 apresentou a maior mediana (20,50ppb), seguida de reduções até o grupo 5 (14,47ppb), cujo primeiro quartil esteve entre os mais baixos. O grupo 6 interrompeu essa tendência, com mediana de 16,60ppb. As amplitudes variaram de 17,6ppb (grupo 6) a 25,5ppb (grupo 3), indicando grande variabilidade interna em todos os grupos (Tabela 1).

## Discussão

Os resultados revelam um quadro complexo de exposições ambientais na Região Metropolitana de São Paulo, com iniquidades que variam conforme o indicador analisado. Embora o IPVS ordene a vulnerabilidade, a vegetação, a temperatura e a poluição não seguiram tendência única, refletindo a diversidade territorial e socioambiental da região.

A cobertura vegetal não apresentou relação linear com a vulnerabilidade social, já que as medianas variaram pouco entre os grupos. O grupo 5 apresentou a maior mediana e a presença de setores em faixas altas do NDVI, possivelmente por sua localização periurbana com a maior presença de vegetação ^12^. Os grupos 2 e 6 registraram as menores medianas e distribuições semelhantes, indicando exposições parecidas entre esses grupos. Todos os grupos mostraram heterogeneidade interna, reforçando que o acesso às áreas verdes na Região Metropolitana de São Paulo é desigual entre e dentro de cada estrato social. A elevada vegetação observada no grupo 5, decorrente da ocupação de áreas próximas a zonas protegidas, muitas vezes não vem acompanhada de infraestrutura adequada, o que reduz o seu potencial de uso para recreação e lazer ^12^. Nas regiões centrais, onde predominam grupos de menor vulnerabilidade, verifica-se que há maior disponibilidade de parques e partes do território com uma distribuição mais equilibrada das áreas verdes no espaço urbano ^12^.

A análise da TST mostra diferença entre o grupo 1, com menor mediana; e os grupos 2, 3, 4 e 6, com valores mais altos e distribuições concentradas em faixas superiores, indicando maior exposição ao calor. O grupo 2 se destacou pela maior mediana e menor dispersão, possivelmente ligada à urbanização marcada por baixa vegetação ^13^. O grupo 5 se aproximou do grupo 1 na mediana, mas incluiu setores com menores temperaturas, possivelmente associados à maior presença de áreas verdes. A exposição ao calor urbano incide sobre todos os estratos sociais, mas os recursos de proteção são desiguais, incluindo desde a qualidade habitacional e o uso de climatização até a possibilidade de evitar deslocamentos nos horários de pico de calor ^14^.

Quanto ao NO_2_, observou-se padrão diferente ao das demais variáveis, com maiores concentrações nos grupos de menor vulnerabilidade, localizados próximos dos principais eixos viários e vias com alto fluxo veicular, em bairros centrais e economicamente dinâmicos, locais de grande emissão de poluentes em áreas urbanas ^13^. A variabilidade interna em todos os grupos reforça que nenhum nível está isento da exposição aos poluentes atmosféricos. Frente ao limite anual recomendado pela Organização Mundial da Saúde (aproximadamente 5ppb) ^15^, a maioria da população da Região Metropolitana de São Paulo se encontra exposta acima do aceitável, um quadro crítico de risco para a saúde. No grupo 6, o valor mínimo observado (6,93ppb) indica que toda a sua população está exposta a níveis superiores ao limite. As altas concentrações nas áreas ricas expõem a contradição urbana: territórios valorizados concentram infraestrutura e empregos, mas também maior poluição. Ainda assim, as classes mais altas permanecem nas centralidades e apresentam maior capacidade de reduzir seus efeitos, em especial por realizarem deslocamentos mais curtos, diminuindo o tempo de exposição, além de contarem com melhor estado de saúde e acesso a cuidados médicos ^16^.

Entre os pontos fortes deste estudo, destaca-se o uso de múltiplos indicadores ambientais em alta resolução combinados a um indicador de vulnerabilidade social, permitindo identificar desigualdades socioambientais em microescala e heterogeneidades intraurbanas relevantes à saúde pública e justiça ambiental. A utilização do NDVI neste trabalho se justifica por sua ampla consolidação em estudos de áreas verdes ^17^. Já a TST, embora não represente a temperatura do ar, é reconhecida como medida útil da exposição ao calor urbano ^18^. Entre as limitações, a estimativa de NO_2_ baseou-se em modelo calibrado sobretudo com dados da Europa e da América do Norte, implicando potencial incerteza para as estimativas na América do Sul ^9^. O uso do IPVS de 2010 pode não refletir a situação atual, assim como as estimativas de exposição ambiental aproximadas ao período de sua elaboração, mas possibilita interpretar o contexto da época e servir de referência para comparações futuras com dados atualizados. Por fim, as estimativas de exposição foram atribuídas apenas ao local de residência, desconsiderando deslocamentos diários que podem alterar os níveis de contato com áreas verdes, calor e poluição, resultando em possíveis erros de medição.

Os achados mostram que as exposições ambientais não se distribuem uniformemente, coexistindo níveis distintos de risco entre grupos sociais e áreas urbanas. Embora este estudo não tenha analisado o recorte de raça/cor, a literatura sobre justiça ambiental alerta que tais vulnerabilizações frequentemente se sobrepõem a estruturas de racismo ambiental ^19^, demandando que futuras investigações abordem essa interseccionalidade.

Para solucionar essas questões é essencial identificar locais de alta vulnerabilização social que também apresentem condições ambientais críticas, como menor cobertura vegetal e níveis elevados de TST e NO_2_, pois são esses territórios que mais se beneficiam de políticas públicas direcionadas. Tais ações devem considerar as especificidades territoriais, ampliando o acesso a áreas verdes, mitigando ilhas de calor e reduzindo a poluição. O uso de indicadores ambientais associados a medidas de vulnerabilidade, como o IPVS, é um modo útil para identificar desigualdades urbanas. No entanto, a compreensão das iniquidades exige atenção à heterogeneidade intraurbana, à sobreposição de exposições em um mesmo território e à interação entre fatores ambientais, sociais e institucionais que moldam os riscos e oportunidades à saúde ^20^.

## Conclusão

O estudo mostrou que a distribuição da vegetação, da temperatura de superfície e da concentração de NO_2_ na Região Metropolitana de São Paulo não segue padrão linear em relação ao IPVS. Grupos populacionais distintos enfrentam condições ambientais diversas: maior cobertura vegetal em áreas de alta vulnerabilidade, temperaturas elevadas tanto em territórios muito vulnertabilizados quanto em áreas de baixa vulnerabilidade, e maiores níveis de poluição em regiões menos vulnerabilizadas. A variabilidade interna de cada grupo evidencia que as desigualdades ambientais também se expressam intragrupo, revelando heterogeneidade socioambiental no espaço urbano.

Do ponto de vista da saúde urbana e coletiva, os resultados são relevantes para analisar desigualdades de exposição ambiental e identificar áreas socioambientalmente críticas. Políticas públicas guiadas pela justiça ambiental devem priorizar esses territórios, expandindo infraestrutura verde, mitigando ilhas de calor e reduzindo a poluição do ar, com ações urbanísticas articuladas às de saúde pública voltadas à minimização dos impactos negativos à saúde. Tais medidas são fundamentais para reduzir desigualdades, melhorar a qualidade de vida e promover cidades mais equitativas e saudáveis.

## Data Availability

Os dados de pesquisa estão disponíveis mediante solicitação ao autor de correspondência.
